# Double pyramidal lobe of the thyroid gland: report of two consecutive cases with surgical and oncologic implications

**DOI:** 10.1093/jscr/rjag049

**Published:** 2026-02-08

**Authors:** Enver Tansu Ağar, Cemil Yüksel

**Affiliations:** Department of Surgical Oncology, University of Health Sciences Mersin Training and Research Hospital, Korukent Mah. 96015 Sok., Mersin Entegre Sağlık Kampüsü, 33240 Toroslar, Mersin, Türkiye; Department of Surgical Oncology, University of Health Sciences Mersin Training and Research Hospital, Korukent Mah. 96015 Sok., Mersin Entegre Sağlık Kampüsü, 33240 Toroslar, Mersin, Türkiye

**Keywords:** double pyramidal lobe, thyroid gland, papillary thyroid carcinoma, anatomical variation, thyroidectomy

## Abstract

The pyramidal lobe (PL) of the thyroid gland is a common embryological remnant; however, the presence of double PLs is an exceptionally rare anatomical variation. Failure to recognize this anomaly may result in incomplete thyroidectomy, particularly in patients with thyroid malignancy. We report two consecutive cases of double PL identified intraoperatively within a 2-day interval at a single center. The index case was a 51-year-old woman operated on for a suspicious thyroid nodule, with final pathology revealing classic variant papillary thyroid carcinoma (2.3 cm) with central lymph node metastases (pT2N1a, AJCC 8th edition). Two distinct PLs arising from the isthmus were identified and completely excised. Two days later, a 55-year-old man with multinodular goiter and a history of asthma underwent thyroidectomy, during which an identical double PL configuration was detected and removed. In both cases, preoperative ultrasonography failed to identify the PLs. The occurrence of two consecutive cases suggests that double PL may be underrecognized rather than truly exceptional. Systematic exploration of the prelaryngeal region is essential to ensure complete thyroidectomy and optimal oncologic outcomes.

## Introduction

The thyroid gland typically consists of two lateral lobes connected by an isthmus. A pyramidal lobe (PL), derived from the thyroglossal duct remnant, may extend superiorly toward the hyoid bone and is reported in up to 40%–65% of individuals. Because of this frequency, the PL is often regarded as a common anatomical component [1].

In contrast, the presence of two PLs, termed double pyramidal lobe (DPL), is exceedingly rare. Only a limited number of cases have been reported in the literature, most as isolated findings. The clinical importance of this variation lies in the risk of residual thyroid tissue after surgery, particularly relevant in Graves’ disease and differentiated thyroid carcinoma [2].

We present two consecutive surgically documented cases of DPL, one associated with papillary thyroid carcinoma and the other with benign multinodular goiter, emphasizing the surgical and oncologic implications of this rare anatomical variant.

## Case presentation

### Case 1 (index case)

A 51-year-old woman with chronic kidney disease (non-dialysis) and hypertension was referred for evaluation of a thyroid nodule. Her surgical history included cervical spine surgery, multiple renal stone operations, and cesarean sections. Family history revealed multiple thyroid surgeries for benign disease.

#### Preoperative evaluation

Neck ultrasonography demonstrated a 19 × 13 mm hypoechoic nodule with lobulated margins and internal microcalcifications in the posterior mid-zone of the left thyroid lobe [Thyroid Imaging Reporting and Data System (TI-RADS 4–5)]. Fine-needle aspiration biopsy reported suspicion for malignancy; papillary carcinoma could not be excluded. No PL was identified on imaging.

#### Surgery

**Figure 1:**
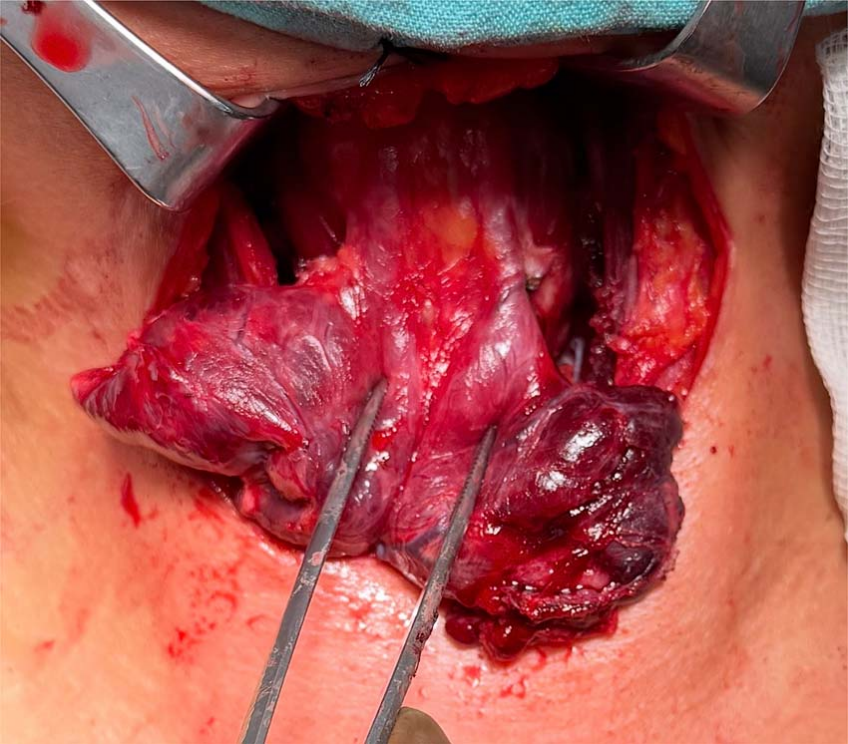
Intraoperative view demonstrating two PLs arising from the superior aspect of the thyroid isthmus and extending cranially toward the infrahyoid region.

Total thyroidectomy with central lymph node dissection was performed. During routine cervical exploration, two distinct PLs arising from the superior aspect of the isthmus and extending cranially toward the infrahyoid region were incidentally identified and completely excised (Fig. 1).

#### Histopathology

- Papillary thyroid carcinoma, classic variant, 2.3 cm, left lobe
- Extrathyroidal extension into surrounding adipose tissue
- No vascular invasion; perineural invasion present
- Metastatic involvement of central lymph nodes (largest 2.5 cm)
- Final stage: pT2N1a (The American Joint Committee on Cancer 8th edition)
- PLs: thyroid tissue without separate malignant focus

The postoperative course was uneventful.

### Case 2

A 55-year-old man with a history of asthma underwent thyroidectomy for multinodular goiter. He had no history of thyroid malignancy.

#### Preoperative evaluation

Neck ultrasonography revealed an enlarged thyroid gland with heterogeneous parenchyma and multiple solid nodules (largest 30 mm in the right lobe and 17 mm in the left lobe). Bilateral cervical lymph nodes were reactive in appearance. No PL was reported on imaging.

#### Surgery

**Figure 2:**
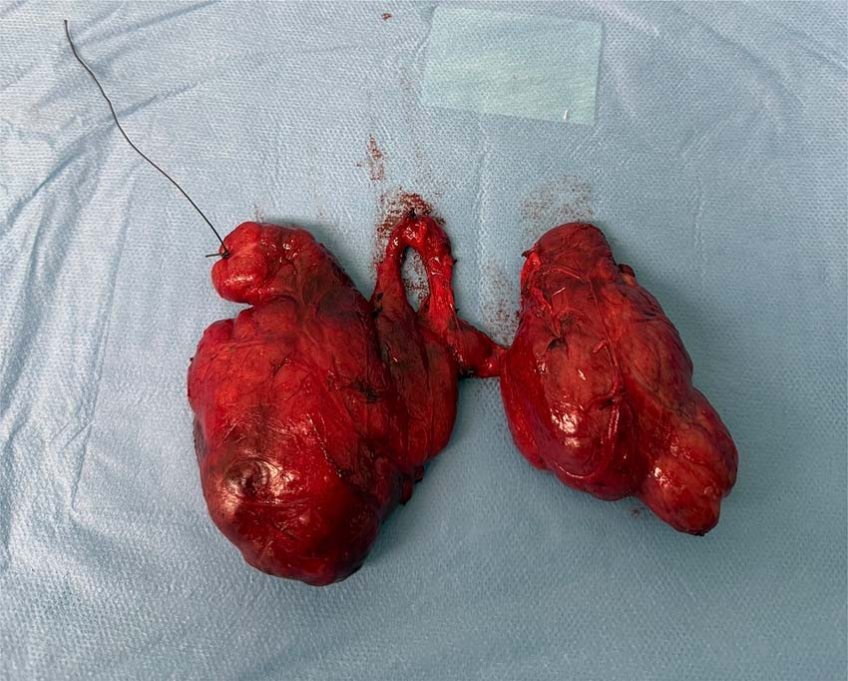
Macroscopic view of the excised thyroid specimen showing two distinct PLs connected to the isthmus by narrow stalks.

**Figure 3:**
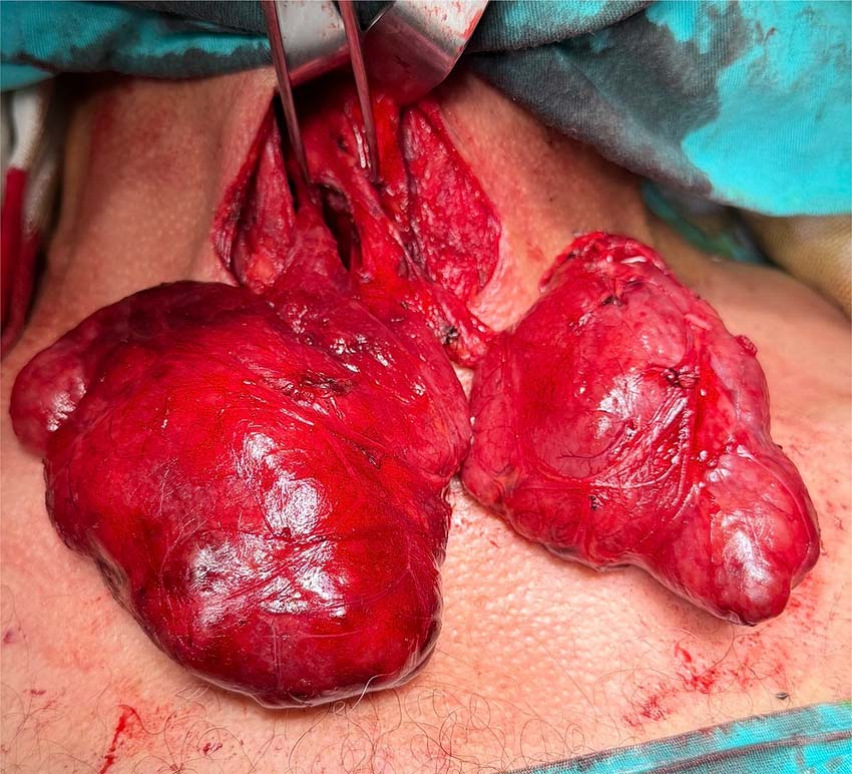
Preoperative view of double PL.

During routine cervical exploration, two distinct PLs arising from the superior aspect of the isthmus were clearly identified. Both extended cranially and showed macroscopic features identical to normal thyroid tissue. They were completely excised en bloc with the thyroid specimen (Figs 2 and 3).

#### Histopathology

- Lymphocytic thyroiditis
- Multinodular colloidal goiter
- No malignancy identified

The postoperative course was uneventful.

## Discussion

Anatomical variations of the thyroid gland are frequently encountered during surgery, with the PL being one of the most common [3]. Although a single PL is present in a substantial proportion of the population, the presence of two separate PLs remains exceedingly uncommon. This rarity is reflected by the very limited number of published reports, most of which describe isolated cases rather than systematic observations.

The PL originates from remnants of the thyroglossal duct, and its development is influenced by the degree and pattern of ductal regression during embryogenesis [4]. In cases of double PL, persistence of thyroid tissue along more than one tract may result in two distinct cranial extensions arising from the thyroid gland. Such a configuration is not routinely anticipated during surgery and may therefore be overlooked unless specifically sought.

From a surgical standpoint, the importance of PLs lies in the fact that they contain functional thyroid tissue. Consequently, any disease affecting the thyroid gland may also involve these extensions. While diffuse thyroid disorders such as multinodular goiter or autoimmune thyroiditis are more likely to involve pyramidal tissue, malignant involvement has also been reported [5]. In the present series, the identification of double PLs in both a patient with papillary thyroid carcinoma and another with benign multinodular goiter emphasizes that this anatomical variation is not disease-specific and should be considered during all thyroidectomies.

One of the most notable findings in our report is the occurrence of two consecutive cases within a short time interval. This observation raises the possibility that double PL is not as exceptionally rare as the literature suggests, but rather underdiagnosed due to lack of routine inspection of the prelaryngeal region. In both of our patients, preoperative ultrasonography failed to detect the PLs, highlighting the limitations of imaging techniques in identifying thin or atypically positioned thyroid extensions.

Failure to recognize and excise PLs may have significant clinical consequences. Residual thyroid tissue following total thyroidectomy can lead to recurrence of benign disease and may present years later as a midline cervical mass [6]. In patients with differentiated thyroid carcinoma, remnant pyramidal tissue may compromise oncologic completeness and interfere with postoperative radioactive iodine therapy by acting as a competing site for iodine uptake. Furthermore, secondary surgery in the central neck carries an increased risk of complications compared to meticulous primary resection.

The findings of this report support the concept that systematic exploration of the prelaryngeal and supraisthmic region should be an integral part of thyroid surgery. Identification and complete excision of all thyroid tissue, including PLs, are essential to optimize surgical outcomes and reduce the risk of recurrence.

In summary, double PL represents a rare but clinically relevant anatomical variation. The presentation of two consecutive cases underscores the need for heightened surgical awareness and suggests that the true incidence of this anomaly may be underestimated. These findings suggest that double PL should be actively searched for during every thyroidectomy, regardless of the underlying pathology.

## Key points

- Double pyramidal lobe (PL) is an extremely rare thyroid anatomical variation
- Two consecutive cases were identified within a short time interval
- One case was associated with papillary thyroid carcinoma and nodal metastasis
- Preoperative imaging failed to identify the PLs in both patients
- Systematic prelaryngeal exploration is crucial during thyroidectomy
